# Counterfactual Reasoning as the Missing Link Between Statistical AI and Human Cognition: A Cognitive Science Perspective

**DOI:** 10.3390/bs16060907

**Published:** 2026-06-03

**Authors:** Piercesare Grimaldi

**Affiliations:** Department of Life Sciences, Health and Health Professions, Link Campus University, 00165 Rome, Italy; p.grimaldi@unilink.it

**Keywords:** counterfactual reasoning, causal inference, large language models, NeuroAI, Default Mode Network, Pearl’s Ladder of Causation, cognitive neuroscience, causal world models

## Abstract

Text-only large language models (LLMs) used as general-purpose reasoners achieve remarkable performance on linguistic and reasoning tasks yet remain limited on intervention-sensitive and individual-level counterfactual reasoning. This article offers a cross-level synthesis linking formal causal competence, cognitive simulation, neural implementation, and artificial intelligence (AI) design criteria. Drawing on formal causal inference theory, developmental cognitive science, and cognitive neuroscience, we first characterize the three levels of causal cognition—association, intervention, and counterfactual reasoning—and show why each is formally irreducible to the one below it. We then discuss concrete, domain-specific categories of LLM failure that are consistent with the absence of a structured causal model capable of supporting individual-level counterfactual invariance. We review neuroscientific evidence for component processes relevant to counterfactual cognition—including episodic construction, fictive evaluation, and internally generated scenario simulation—with the Default Mode Network and hippocampal systems playing important but not exclusive roles. Finally, we propose that the most productive path forward is not to implement Pearl’s formal rules in AI systems, but to study how the brain approximates counterfactual reasoning without ever following those rules explicitly—just as neuroscience has historically provided useful constraints, architectural motifs, and evaluation criteria for AI research.

## 1. Introduction

In a February 2026 interview with La Repubblica, Nobel laureate Giorgio Parisi offered a precise verdict on the current state of artificial intelligence (AI): today’s systems can interpret text, hold conversations, and answer questions—but they do not go beyond language, and their knowledge of the physical world around us is said to be worse than that of a mouse. Asked what must be done to truly understand artificial intelligence, he replied: “We need to understand the brain. Too much of its functioning still escapes us.” He then emphasized that AI reproduces language, which developed only recently in evolutionary terms, whereas the mammalian brain has a far deeper evolutionary history (Allman, 2000; Dusi, 2026; Kaas, 2013). The entire pre-linguistic part of our intelligence eludes us. Studying it is not easy, but it offers enormous possibilities for developing the AI of the future. Current AI systems have built their architecture on the tip of the iceberg, while the mass beneath remains uncharted.

This distinction is not merely philosophical. It has direct, measurable consequences for the behavior of AI systems in the wild: adversarial fragility (Wallace et al., 2019; Zou et al., 2023), confident hallucination in applied generation tasks (Zhang et al., 2025), and instability on causal and counterfactual reasoning benchmarks (Jin et al., 2024; Joshi et al., 2024; Zečević et al., 2023). These phenomena should not be treated only as engineering bugs to be patched by scale; they may also reflect a deeper representational limitation. Biological cognition appears to address related problems not merely by accumulating more data, but by constructing and revising structured models of action, outcome, and context (Gopnik, 2009; Johnson-Laird, 1995; Meltzoff, 1995).

This article does not address artificial intelligence as a whole. Its primary target is large language models (LLMs) used as general-purpose reasoners, especially text-only systems operating without explicit causal models or direct interaction with the world. The central claim is that such systems have not yet demonstrated the representational stability required for robust intervention-sensitive and individual-level counterfactual reasoning. This is not a new observation (Zečević et al., 2023), but its implications for the cognitive science of AI design remain underexplored. I refer to these models below as “text-only LLM reasoners.”

Crucially, however, we do not argue that AI should implement Pearl’s Ladder formally. The brain almost certainly does not. What the brain does is solve the problem that Pearl’s Ladder describes—robustly, efficiently, and without explicit calculus.

This inference is supported by a well-established precedent in cognitive science. Kahneman et al.’s foundational work on heuristics and biases demonstrated that human reasoning systematically departs from the normative rules of classical logic and probability theory—yet remains adaptive and ecologically valid (Kahneman, 2013; Kahneman & Miller, 1986). If the brain does not implement the formal axioms of probability, there is little principled reason to expect it to implement Pearl’s do-calculus either. What the brain appears to implement, instead, is a *functional analog* of causal inference: a set of evolved, experience-dependent mechanisms that approximate the computational goals described by Pearl’s framework. The normative framework thus serves as a *computational-level description*, *in the sense of* *Marr* (1982), of what the brain is trying to solve, not as a blueprint for how it solves it.

This dissociation is the central lesson for AI design: the goal is not to encode causal formalism into neural networks, but to understand how biological systems approximate counterfactual reasoning heuristically, and to replicate that approximation. This is consistent with a broader lesson from AI history: biological systems have often provided productive constraints on AI architecture, a point developed further in Section 5.

We proceed as follows. Section 2 formalizes the three levels of causal cognition and their cognitive requirements. Section 3 discusses domain-specific categories of LLM failure that are consistent with the absence of a structured causal model capable of supporting counterfactual invariance. Section 4 reviews the cognitive science of counterfactual reasoning, from its developmental origins to its neural implementation. Section 5 discusses the NeuroAI research program as a principled path forward. Section 6 discusses implications and open questions. Section 7 concludes.

The original contribution of this Viewpoint is to propose counterfactual invariance as a cross-level criterion linking formal causal inference, mental-model-based cognitive simulation, neural scenario construction, and concrete NeuroAI design pathways.

## 2. The Ladder of Causation: Three Irreducible Cognitive Levels

Judea Pearl’s Ladder of Causation (Pearl, 2000; Pearl & Mackenzie, 2020) provides the most rigorous available taxonomy of causal cognition. Its three rungs—association, intervention, and counterfactual reasoning—are not merely ordered by difficulty. Each is formally incommensurable with the one below it: no amount of data at level *n* is sufficient to answer a question at level *n* + 1.

### 2.1. Level 1: Association

Formally expressed as *P*(*Y | X*)—the conditional probability of *Y* given observation of *X*—association is the detection of statistical regularities in observed data. This is the level at which text-only LLM reasoners primarily operate. The paradigmatic example from cognitive science is the notorious ice cream–drowning correlation: both rise together in summer, yet ice cream consumption does not cause drowning. A system operating at Level 1 cannot distinguish genuine causes from confounds (Pearl, 2000).

### 2.2. Level 2: Intervention

Formalized as *P*(*Y |* do(*X*)), the probability of *Y* given that we actively set *X*, rather than merely observe it—intervention requires a qualitatively different cognitive operation. The do() operator disconnects *X* from its natural causal parents, mimicking the structure of a randomized experiment. This is the level at which a clinician reasons when asking: “If I administer this drug, what will happen to the patient’s blood pressure?” as opposed to merely noting that patients who receive it tend to have lower blood pressure.

Crucially, intervention cannot be derived from association data alone (Hernán & Robins, 2024). No amount of observational data, however large, licenses the inference from correlation to the consequence of manipulation. This is why text-only LLMs trained primarily on observed linguistic co-occurrences cannot reliably answer causal questions when they lack an explicit causal model, embodied interaction, or external world model (Pearl, 2000).

### 2.3. Level 3: Counterfactual Reasoning—Imagining

The highest rung is formalized as P(*Y_x_* | *X = x′*, *Y* = *y*)—the probability that *Y* would have taken value *Y_x_*, had *X* been *x′*, given that we in fact observed *X* = *x* and *Y* = *y*. This requires reasoning about a specific individual in a specific situation that did not occur. A judge asking “but for the defendant’s action, would the harm have occurred?” is operating at Level 3. A physician asking “would this patient have survived without the treatment?” is operating at Level 3.

What makes this level cognitively special is that it requires simultaneously holding in mind the actual world and a hypothetical alternative, mapping the individual’s specific circumstances across both, and reasoning about the structural equations that govern the causal mechanisms—all while suppressing the pull of what actually happened.

Critically, what is missing is not counterfactual language, but a structured representational substrate that encodes individual entities, their causal dependencies, and preserves identity invariance (Pearl, 2000).

## 3. Where Text-Only LLM Reasoners Fail: Concrete Instances of Counterfactual Deficit

These failure modes are not individually diagnostic; their relevance is cumulative: together, they motivate the need for controlled tests of counterfactual invariance, such as the diagnostic proposed in Section 3.4.

Some have argued that current LLMs approximate Level 2 reasoning on familiar causal structures (Jin et al., 2024), but it is possible that this reflects pattern-matching over causally described text rather than genuine interventional reasoning—these models may talk causality but are not causal (Zečević et al., 2023): performance degrades systematically when the causal structure is novel or the query requires manipulation of an explicit directed acyclic graph (DAG).

### 3.1. Adversarial Fragility

Adversarial examples, as shown by Goodfellow et al. (2014), reveal that small perturbations can cause high-confidence errors. Adversarial fragility is not, by itself, diagnostic of absent counterfactual reasoning. However, it illustrates a broader concern relevant to the present argument: model outputs can depend on surface-level perturbations that are causally irrelevant to the underlying situation. For this reason, adversarial fragility is best treated as background evidence for representational instability, not as direct evidence of Level 3 failure.

### 3.2. Collider Reasoning

In scenarios involving collider bias, LLMs often reproduce surface correlations rather than identifying the underlying causal structure. Unless guided, they fail to distinguish conditioning from intervention (in the sense of Judea Pearl), indicating a lack of spontaneous interventional reasoning (Joshi et al., 2024; Zečević et al., 2023).

This is primarily a Level 2 failure because it concerns the distinction between observation, conditioning, and intervention. It should therefore not be conflated with Level 3 individual counterfactual inference. Its relevance is that robust Level 3 reasoning presupposes, at minimum, stable Level 2 competence.

### 3.3. Individual Counterfactuals

When asked whether a specific individual would have had a different outcome under an alternative intervention, LLMs default to population-level statistics. They lack a mechanism to anchor counterfactuals to a fixed individual, thereby conflating average treatment effects with individual causal effects (Pearl, 2000; Pearl & Mackenzie, 2020). This limitation is consistent with recent evidence that LLMs struggle with counterfactual and interventional reasoning beyond associative patterns.

### 3.4. Counterfactual Invariance Under Causally Equivalent Reformulations

A genuine causal reasoner should preserve its answer when the linguistic surface of a question changes but the underlying causal structure remains constant. This is particularly important for counterfactual questions, where the system must preserve the identity of an individual across actual and hypothetical worlds while selectively modifying one causal variable. The relevant issue is not whether an LLM can answer a simple counterfactual example correctly. Often it can. The stronger diagnostic question is whether the answer remains stable when the same individual case is presented under different contextual framings, reordered information, or irrelevant distracting details. In such cases, LLMs may shift from the relevant causal structure to surface associations, narrative plausibility, or population-level regularities. Thus, the critical weakness is not occasional incorrectness, but instability: a system with robust Level 3 competence should preserve the same answer across causally equivalent reformulations and change it only when the causal structure itself changes. Systematic divergence under such controlled reformulations would therefore provide stronger evidence of counterfactual instability than failure on any single example.

## 4. The Cognitive Science of Counterfactual Reasoning

Counterfactual reasoning, in its fully explicit human form, is not reducible to a single neural mechanism. It appears to depend on several component processes that are older than language: intention inference, action–outcome learning, episodic construction, fictive evaluation, and model updating (Fitch, 2010; Friston, 2010; Gopnik, 2009; Meltzoff, 1995).

Causal reasoning precedes language acquisition. This is an important fact: in Meltzoff’s (1995) classic experiment, 18-month-old infants, who cannot yet produce full sentences, observe an adult repeatedly attempting and failing to hand them an object. The infants consistently reach out to complete the adult’s intended action, demonstrating an intuitive understanding of the immediate physical cause. This shows that intention inference is a component process relevant to later counterfactual reasoning, although it should not be treated as direct evidence for formal counterfactual inference.

Gopnik et al. (2001) blicket detector experiments extend this picture. Children aged 2–4, with no instruction, observe a machine that activates for some objects but not others, then spontaneously update their causal model when a new object activates the machine unexpectedly. They are not memorizing input–output pairs; they are constructing and revising causal hypotheses in real time (Gopnik, 2009).

LLMs invert this developmental logic: they are trained on language, from which they attempt to extract causal structure—precisely backwards from the order in which cognition evolved and develops.

### 4.1. Neural Component Processes Supporting Counterfactual Thought

Converging findings from neuroscience and developmental psychology identify a set of component processes that may constitute the building blocks of counterfactual cognition. Converging evidence from studies on imagination and episodic simulation has consistently implicated the Default Mode Network (DMN) in internally generated alternatives to reality (Barbey et al., 2009; De Brigard et al., 2013; Schacter et al., 2012; Van Hoeck et al., 2013). While episodic simulation and counterfactual reasoning are not identical, they likely share core generative mechanisms. Building on this work, we propose that the DMN, comprising core regions such as the medial prefrontal cortex and posterior cingulate cortex/precuneus, along with lateral parietal areas (including the angular gyrus) and medial temporal lobe structures (including the hippocampus), constitutes an intrinsically organized network that is active during rest and typically suppressed during externally directed task performance (Raichle, 2015).

Functionally, this system may implement generative recombination of structured representations under minimal sensory constraints. Far from being mere idling, DMN activity underlies mental time travel, social simulation, and the generation of counterfactual scenarios (Buckner et al., 2008).

The hippocampus contributes episodic memory reconstruction—not veridical replay but flexible recombination of stored elements into novel configurations. Patients with hippocampal lesions not only lose episodic memory; they lose the ability to imagine future events and construct alternative pasts, supporting a central role for hippocampal mechanisms in scenario construction (Hassabis et al., 2007).

The prefrontal cortex (PFC) contributes to the representation of counterfactual outcome values, including regret and fictive error signals—the subjective evaluation of what might have occurred under alternative choices (Camille et al., 2004; Coricelli et al., 2005). The orbitofrontal cortex provides a complementary contribution by encoding the subjective value of available options within a given task space, including those not ultimately chosen, thereby supporting the comparison processes that give counterfactual representations their behavioral and motivational relevance (Padoa-Schioppa & Assad, 2006).

Taken together, these prefrontal mechanisms support a form of counterfactual evaluation in which alternative possibilities are represented in a sufficiently rich and flexible manner to guide adaptive inference and decision-making, approximating key features of higher-order causal reasoning.

### 4.2. The Role of Mental Models

Johnson-Laird’s theory of mental models provides the cognitive-scientific bridge between formal causal reasoning and biological cognition. In his view, human reasoners do not normally apply formal logic, probability calculus, or structural causal equations explicitly. Rather, they construct internal models of possible states of affairs and evaluate conclusions by inspecting, modifying, and comparing these models. A mental model can be understood as a token-based representation of a scenario: a temporary configuration of entities, relations, events, and constraints that stands for a possible situation (Johnson-Laird, 1995; Kahneman & Miller, 1986).

Counterfactual reasoning, in this framework, requires the construction of at least two related tokens: one representing the actual situation and another representing a minimally modified alternative. For example, the question “Would the patient have survived without the treatment?” requires preserving the identity of the patient and the relevant background conditions while selectively altering one element—the administration of treatment—and then evaluating the downstream consequences of that alteration. The operation is therefore not merely linguistic substitution. It involves maintaining continuity between actual and hypothetical scenarios while manipulating a restricted part of the represented situation.

This differs from Pearl’s formal causal model. In Pearl’s framework, counterfactual inference requires a structural causal model: a set of variables connected by structural equations, together with a defined intervention operation that modifies one part of the model while holding the rest of the causal structure fixed. Such a model is formally learnable, manipulable, and mathematically explicit. It specifies what changes, what remains invariant, and how downstream variables are recomputed under intervention.

Mental models are less formal but cognitively plausible approximations of this requirement. They do not require the reasoner to represent a full directed acyclic graph (DAG) or solve structural equations symbolically. Instead, they allow the reasoner to build a small-scale scenario, alter one component, and inspect the resulting implications. This is why counterfactual reasoning is affected by the ease of constructing the alternative model: alternatives that are simple, familiar, or close to the actual event are easier to simulate than remote or structurally complex alternatives.

The contrast is therefore not between “having” and “not having” causal reasoning, but between two levels of description. Pearl’s model specifies the normative structure of the problem: what a system would need to represent to perform valid intervention and counterfactual inference. Johnson-Laird’s theory describes a psychologically plausible mechanism by which biological agents may approximate such reasoning: constructing, modifying, and comparing scenario tokens. This distinction is central to the present argument. The brain need not implement formal causal calculus in order to approximate some of its computational functions. Conversely, an AI system that produces fluent causal explanations does not necessarily possess a manipulable scenario representation capable of preserving entity identity, causal invariance, and selective intervention across possible worlds.

## 5. The NeuroAI Blueprint

The NeuroAI research program, articulated most comprehensively by Zador et al. (2023), argues that the path to more capable AI runs through neuroscience, not through scale alone. The argument is not that the brain should be mimicked superficially, but that it should be studied functionally: it solves a class of problems that AI cannot, and understanding the principles behind that solution is more productive than deriving solutions from abstract formalism. This dissociation between formal description and biological implementation is not a limitation to be explained away; it is the central lesson. Building AI systems that replicate that approximation is a more tractable and more promising program than encoding causal formalism directly.

Convolutional neural networks (CNNs) were influenced by Hubel and Wiesel’s (1959) discovery of hierarchical receptive fields, but also by advances in signal processing and gradient-based optimization. Reinforcement learning has deep roots in control theory and dynamic programming; the link to dopaminergic prediction-error signals (Schultz et al., 1997) came later as a productive biological convergence, not as a derivation. Transformer attention was not derived from prefrontal-parietal circuits, though structural parallels may still suggest useful hypotheses. In each case, biological findings helped identify useful computational constraints or motifs, without serving as a complete derivation of the resulting AI method.

### 5.1. Two Concrete NeuroAI Research Pathways

To make this NeuroAI proposal more operational, we outline two concrete research pathways through which biological principles could inform AI systems capable of more robust intervention and counterfactual reasoning.

#### 5.1.1. DMN-Inspired Generative Modules for Counterfactual Exploration in Transformers

A first pathway concerns the design of auxiliary generative modules inspired by the Default Mode Network. The relevant biological principle is not that the DMN “is” a counterfactual engine in a formal sense, but that it supports internally generated scenario construction, episodic recombination, mental time travel, and the exploration of alternatives under reduced sensory constraint. In computational terms, this suggests an architectural separation between an externally oriented task module and an internally oriented simulation module.

In a transformer-based system, such a module could be implemented as a latent scenario generator operating alongside the main language model. Given an input describing an actual situation, the module would construct alternative latent scenario tokens by selectively modifying one causal variable while preserving entity identity and background constraints. For example, in a clinical counterfactual question, the system would need to preserve the same patient, comorbidities, baseline risk, and temporal context, while altering only the intervention variable. The generated alternatives would then be passed back to the task module for comparison with the actual scenario.

The relevant principles are: generative recombination; partial decoupling from immediate input; identity preservation; and selective intervention. Success would not be measured by fluency of explanation alone, but by performance on controlled tasks requiring invariance across paraphrases, correct distinction between observation and intervention, and stable individual-level counterfactual inference.

#### 5.1.2. In Vitro Neural Cultures and Causal Sequence Learning on Multi-Electrode Arrays

A second pathway concerns simplified biological systems, such as in vitro neural cultures grown on multi-electrode arrays (MEAs). These preparations do not model human counterfactual reasoning directly. Their value is more limited and mechanistic: they may allow researchers to study how recurrent biological networks learn temporal contingencies, update predictions after perturbation, and reorganize activity after changes in input–outcome structure.

A possible experiment would expose a neural culture to repeated stimulation sequences in which specific stimulation patterns predict later sensory or reward-like feedback. Once a stable sequence–outcome mapping has been learned, the experimenter could introduce controlled perturbations: omitting one element, reversing the order, substituting an intermediate event, or delivering feedback inconsistent with the learned sequence. The main measurements would be changes in population dynamics, prediction-error-like responses, recovery trajectories, and the emergence of separable neural states for expected versus violated sequences.

The AI-relevant output would be computational, not anatomical: local updating after causal violation, recurrent stabilization of learned sequences, sensitivity to temporal order, and rapid reconfiguration after perturbation. These principles could inform recurrent memory modules, predictive state representations, or world-model components that update internal transition models when the expected causal sequence is disrupted.

This pathway is therefore best understood as a bridge from biological sequence learning to AI model revision. It would address Level 2 competence more directly than Level 3 counterfactual reasoning: the system learns that manipulating a sequence element changes later outcomes. Only after such intervention-sensitive sequence models are established would it become meaningful to ask whether the system can support Level 3 questions, such as what would have happened in a specific past trial had one element of the sequence been different.

These two pathways illustrate the broader claim of this article. Neuroscience should not be invoked as a vague source of inspiration. It should be used to identify specific computational operations—scenario generation, entity preservation, selective perturbation, prediction updating, and model revision—that can be implemented and tested in artificial systems.

Operationally, Level 2 and Level 3 should be evaluated with different tasks. Level 2 competence can be tested in humans, animals, neural cultures, or artificial agents using intervention tasks in which the system must distinguish observing X from actively setting X and predict the downstream consequence of that manipulation. Level 3 competence requires a stricter test: the system must reason about a specific individual or trial, preserve its identity and background conditions, selectively alter one causal variable, and produce a stable prediction across causally equivalent reformulations. In biological research, humans and non-human animals can be tested with behavioral paradigms involving action–outcome contingencies, omitted actions, and altered feedback; simplified preparations such as MEA cultures are better suited to studying Level 2 sequence perturbation and model revision rather than full Level 3 counterfactual inference. In AI, success should be evaluated not by fluent explanation alone, but by invariance across paraphrases, correct observation/intervention distinction, preservation of entity identity, and appropriate revision after selective causal perturbation.

## 6. Open Questions and Implications for AI Design

Several implications follow from the analysis above. First, the standard scaling hypothesis—that sufficiently large language models trained on sufficiently large text corpora will eventually exhibit counterfactual reasoning—remains unsupported in its strong form. Developmental evidence suggests that causal cognition emerges through active exploration, intervention, and model revision rather than through passive exposure to linguistic descriptions alone (Gopnik, 2009; Gopnik et al., 2001). By contrast, text-only LLMs are trained primarily on linguistic traces of such reasoning. For this reason, their causal competence should not be inferred from fluent causal language alone, but must be evaluated using tasks that distinguish associative prediction from intervention-sensitive and individual-level counterfactual inference.

Second, the most tractable research program may involve simpler biological systems. The basic mechanisms of causal cognition are present, in simpler form, in non-human animals—making them accessible to the mechanistic circuit-level analysis that is not yet possible in the human brain. In vitro neural network models on multi-electrode arrays, of the kind being developed in the context of biocomputing research, may offer particularly direct access to the cellular and network-level mechanisms of causal inference and model revision (Kagan et al., 2022).

Third, the implications for AI safety and accountability are substantial. Systems that reason counterfactually are, in principle, more amenable to post hoc explanation than associative systems: they can be asked, “why did you reach this conclusion, and would you have reached it under different conditions?” Systems may produce fluent explanations without possessing a causal model. The relevant test is whether their explanations remain stable under causally irrelevant reformulations and change appropriately under selective causal interventions.

## 7. Conclusions

The argument advanced here can be stated compactly. Text-only LLMs used as general-purpose reasoners do not robustly implement Level 2 reasoning on novel causal structures and have not been shown to support stable Level 3 individual counterfactual inference: they detect statistical associations with extraordinary efficiency, but they have not yet been shown to support stable, identity-preserving counterfactual simulation under controlled reformulation. Human cognition appears capable of Level 3 reasoning in many ordinary contexts, most likely not through formal probabilistic calculus, but through a simulation-based, heuristic capacity that is evolutionarily ancient, developmentally early, and neurally distributed.

The gap between these two is not primarily a matter of scale. It is a matter of architectural principle. But the solution is not to implement Pearl’s formal rules in AI systems—the brain does not follow those rules either, and yet it solves the problem. The productive question is not ‘how do we encode the Ladder of Causation into a neural network?’ but ‘how does the brain approximate counterfactual reasoning without explicit formalism, and how do we replicate that approximation?’

Several evidentiary gaps remain open. It is not yet established which neural circuits specifically support individual-level counterfactual inference, as distinct from episodic construction or fictive error processing. It is not yet known whether the component processes identified in developmental and neuroscientific research—intention inference, scenario construction, causal model updating—are sufficient, when combined, to support formal Level 3 competence, or whether additional representational structure is required. And it is not yet clear how the architectural principles outlined in Section 5 can be validated against behavioral criteria that are demonstrably more demanding than fluency. These are the empirical questions that a NeuroAI research program centered on counterfactual reasoning should address.

## Data Availability

Data sharing is not applicable to this article, as no new data were created or analyzed in this study.
